# The Impact of NOACS versus VKAS on Absolute and Relative Cognitive Function Decline Over Time: A Machine Learning Approach

**DOI:** 10.1055/a-2698-3739

**Published:** 2025-09-25

**Authors:** Sergio Ferrantelli, Mario Daidone, Giuseppe Armentaro, Gaetano Pacinella, Stefania Scaglione, Anna M. Ciaccio, Edoardo Pirera, Carlo D. Maida, Giuseppe Miceli, Giuliana Rizzo, Vittoriano Della Corte, Domenico Di Raimondo, Daniele Pastori, Angela Sciacqua, Antonino Tuttolomondo, on behalf of MEDIREG (Mediterranean Research Group)

**Affiliations:** 1 Internal Medicine and Stroke Care Ward, University of Palermo, Palermo, Italy; 2 Department of Health Promotion, Mother and Child Care, Internal Medicine and Medical Specialities, University of Palermo, Palermo, Italy; 3 Department of Medical and Surgical Sciences, University Magna Græcia of Catanzaro, Campus Universitario di Germaneto, Catanzaro, Italy; 4 Department of Clinical Internal, Anesthesiological and Cardiovascular Sciences, Sapienza University of Rome, Rome, Italy; 5 IRCCS Neuromed, Pozzilli, Italy

**Keywords:** atrial fibrillation, cognitive impairment, dementia, machine learning, NOAC, VKA

## Abstract

**Background:**

Atrial fibrillation (AF) is the most common arrhythmia in older adults and is associated with an increased risk of cognitive impairment and dementia, even in patients without prior stroke. Nonvitamin K antagonist oral anticoagulants (NOACs) offer a better safety profile than vitamin K antagonists (VKAs), but their cognitive benefit remains uncertain.

**Aim:**

To assess the impact of NOACs versus VKAs on cognitive decline in elderly AF patients using a machine learning approach.

**Methods:**

This multicenter prospective cohort study included 983 AF outpatients enrolled between 2008 and 2022 at the Geriatrics Department, University of Catanzaro, and the ProMISE Department, University of Palermo. Stroke and bleeding risks were assessed using CHA _2_ DS _2_ -VASc and HAS-BLED scores. Cognitive function was evaluated using the Mini-Mental State Examination (MMSE). Cognitive decline was defined as a decrease in MMSE score between baseline and follow-up. Patients with prior anticoagulant therapy (OAT), severe dementia, or comorbidities affecting cognition were excluded. Multivariable logistic regression and a random forest classifier were used to assess whether anticoagulant type independently predicted cognitive decline. Class imbalance was addressed using both class-weighted learning and the synthetic minority over-sampling technique (SMOTE), with model performance evaluated through repeated stratified cross-validation and threshold optimization.

**Results:**

At baseline, cognitive performance was comparable between groups ( *p* = 0.11). After a mean follow-up of 7.2 ± 3.4 years, MMSE scores declined significantly more in VKA-treated patients (−1.7 vs. −0.3 points, *p* < 0.001). In logistic regression, NOAC use was independently associated with a lower risk of cognitive decline (odds ratio: 0.322; 95% confidence interval: 0.221–0.469; *p* < 0.0001). The random forest classifier achieved a mean cross-validated AUC of 0.8719 (standard deviation: 0.0273) and a test-set AUC of 0.880. Threshold adjustment and SMOTE improved sensitivity (recall increased: 0.47–0.84), with a precision–recall AUC of 0.763. Permutation importance analysis identified “OAT” as the top predictor. Predicted probabilities of cognitive decline were significantly higher in VKA users (median = 0.70) than in NOAC users (median = 0.09), confirmed by a Kolmogorov–Smirnov test (KS = 0.385, *p* < 0.001).

**Conclusion:**

NOAC use is associated with a lower predicted probability of cognitive decline, suggesting potential cognitive benefits over VKAs.

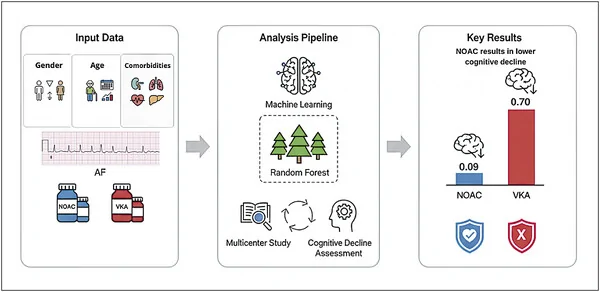

## Introduction

Atrial fibrillation (AF) is the most prevalent cardiac arrhythmia in the elderly population, with a prevalence of 5%, expected to increase 2.3-fold in the future. 1 2 AF is linked to several comorbidities, including heart failure, arterial hypertension (AH), chronic obstructive pulmonary disease (COPD), chronic kidney disease (CKD), and notably cognitive impairment (CoI) and dementia.

The most recent guidelines on the topic from leading scientific societies highlight that the incidence of CoI and dementia is 1.5-fold higher in patients with AF than in the general population, regardless of the presence of previous stroke, and emphasize the importance of continuous assessment of cognitive function in these patients. 2 3 4

It is recognized that AF and CoI share classic cardiovascular risk factors: advanced age, type 2 diabetes mellitus (T2DM), and AH; however, the AF-CoI association persists even after adjusting for known confounders. 5 Additionally, a more rapid cognitive decline is observed in AF patients within 7 years of arrhythmia onset, 6 regardless of stroke history, particularly in those under 70 years. 6 7

Several pathophysiological mechanisms underpin this association. Silent cerebral infarcts (SCI), frequent in AF patients, are significantly associated with CoI and dementia. 8 Microbleeds, often clinically silent, also contribute to CoI and dementia and can be exacerbated by anticoagulant therapy (OAT) used for stroke prevention. This is especially pertinent for patients on vitamin K antagonists (VKAs), as many experience a reduced therapeutic range time (<70%), which increases the risk of microbleeds or SCI. 9 10 In the elderly population, these mechanisms are amplified, promoting atrial thrombosis and thromboembolic complications with evident or silent stroke. 10

Moreover, AF is characterized by an increased ventricular response and impaired atrial contractility, leading to high variability in cerebral blood flow at arteriolar and capillary levels and reduced local autoregulation, potentially causing cerebral hypoperfusion, especially in the temporal lobes. This results in cognitive decline and dementia, including Alzheimer's disease. 11 Consequently, AF patients exhibit reduced total brain volume and hippocampal atrophy compared with those without AF. 12 13

Thromboembolic events clearly contribute to CoI in AF patients, highlighting the importance of preventing such events through OAT. However, the benefits of these drugs remain debated. At the same time, non-VKA oral anticoagulants (NOACs) have a better safety profile and equivalent efficacy compared with VKAs. However, the literature shows no net cognitive function benefit of NOACs over VKAs. 14 15 16 17

The advent of big data, combined with the increasing use of electronic health records (EHRs) and the concurrent development of machine learning algorithms, has empowered healthcare professionals to address population health challenges that were previously deemed impossible. 18 This paradigm shift toward leveraging clinical data at a population level has revolutionized our approach to population-level inferences, enabling the identification and resolution of health issues with enhanced precision and accuracy.

A principal advantage of machine learning lies in its capacity to discern patterns and correlations within complex datasets, yielding valuable insights into disease diagnosis, prognosis, and treatment. Furthermore, automating data analysis through machine learning mitigates human bias and enhances predictive accuracy. The study of big data can yield critical information for decision-making, research, and innovation by unveiling insights and patterns that are challenging to detect in smaller, more structured datasets. 19

### Aim of the Study

To address this, a multicenter prospective cohort study on elderly AF patients aims to assess the impact of NOACs versus VKAs on absolute and relative cognitive function decline over time and determine whether NOAC use is associated with slower cognitive function decline compared with VKA treatment, using a Machine Learning approach.

## Materials and Methods

### Study Setting, Design, and Sample

Between January 2008 and September 2022, a multicenter observational study enrolled all consecutive AF outpatients at the Geriatrics Department, “Magna Graecia” University of Catanzaro, Italy, and the Department of Promoting Health, Maternal–Infant, Excellence, and Internal and Specialized Medicine (ProMISE) G. D'Alessandro, University of Palermo, Italy. Inclusion criteria were a new diagnosis of nonvalvular AF, age ≥65 years, a minimum of 1-year follow-up, and informed consent. Exclusion criteria included valvular diseases, prior oral anticoagulant (OAC) therapy (patients already on OAC before enrollment), severe dementia or Alzheimer's disease, psychiatric comorbidities or drug therapies affecting cognitive function, chronic infectious diseases (e.g., HIV and hepatitis C/B), autoimmune systemic diseases, active cancer, and liver failure (e.g., cirrhosis).

Among 1,366 patients admitted during the study period, 143 were excluded because they were < 65 years old, 101 were on OAT before enrolment, 68 had a mechanical prosthetic valve, 36 had moderate-severe mitral stenosis, 15 had cancer, 10 had liver cirrhosis, and 10 had chronic inflammatory diseases. Therefore, 983 elderly patients were enrolled ( Fig. 1 ).

**Fig. 1:**
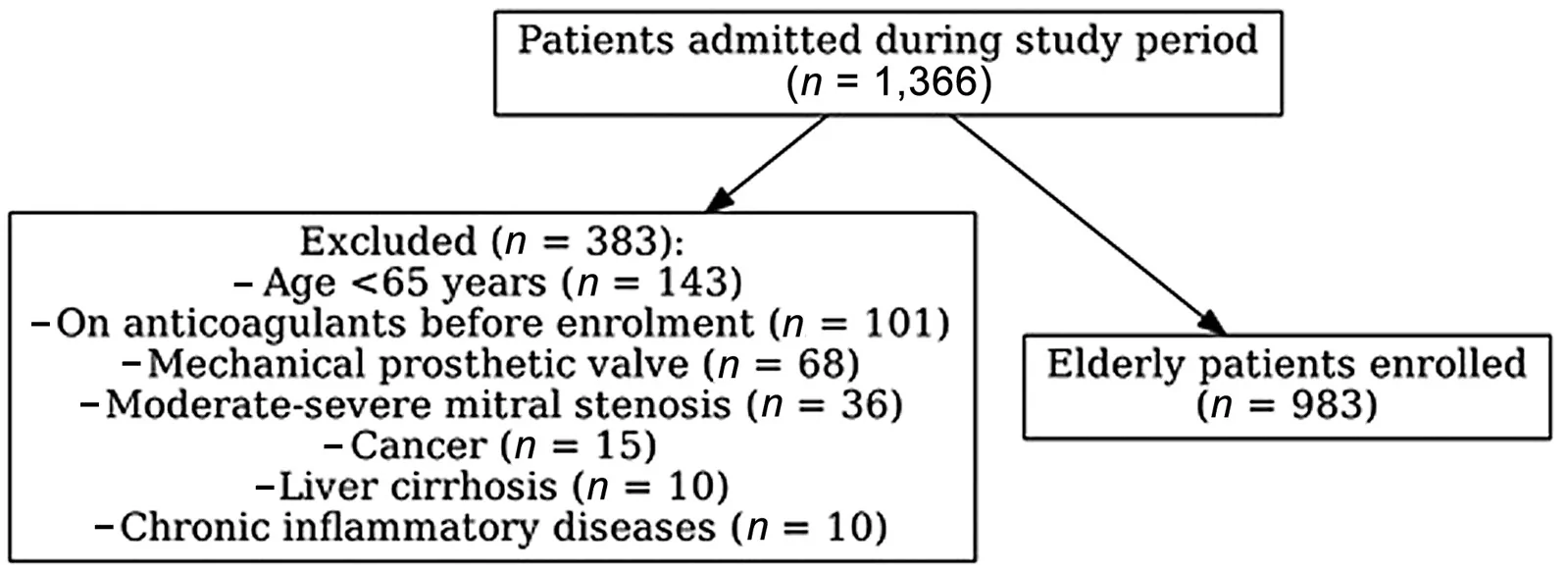
Flowchart of patients admitted to the study and reasons for exclusion.

The local ethical committees granted ethical approval: Comitato Etico Regione Calabria “Area Centro” (protocol number 2012.63 of October 17, 2012) and Comitato Etico Regione Sicilia “Palermo 1” (protocol number 20203.04 of April 19, 2023). The study adhered to the Declaration of Helsinki. Informed written consent was obtained from all participants.

### Clinical Variables at Baseline and Follow-Up

At enrollment and during two follow-up visits, all patients underwent comprehensive medical history reviews, physical examinations, and routine electrocardiography. Measurements of anthropometric and hemodynamic variables (weight, height, BMI, systolic and diastolic blood pressure, and heart rate) were taken. Relevant comorbidities and drug therapies were recorded. Stroke and bleeding risks were assessed using validated tools for AF patients. 2 We defined a HAS-BLED score of 3 or higher as indicating a high bleeding risk. Cognitive function was assessed using the Mini-Mental State Examination (MMSE) at baseline and during follow-up visits. At baseline, cognitive performance was categorized as normal (MMSE > 25), borderline (MMSE = 25), or mild CoI (MMSE < 25). The first follow-up period lasted 3.8 ± 2.6 years (medium period) and the second 7.2 ± 3.4 years (long period).

Cognitive decline was retrospectively defined as the difference in MMSE scores between baseline and the second follow-up visit (mean follow-up: 7.2 ± 3.4 years), without documentation of the exact timing of decline onset. If the MMSE score remained unchanged, indicating a difference of 0, it was not considered to be indicative of cognitive decline.

To address the potential for mortality to act as a competing risk—particularly relevant in a high-risk, elderly population with AF—an exploratory analysis of all-cause mortality during follow-up was conducted.

#### Data Preprocessing

In preparation for the analysis, we employed a rigorous data preprocessing protocol to ensure the accuracy and reliability of the findings. The dataset was initially loaded into a Pandas DataFrame from a CSV file, which facilitated efficient data handling and preliminary exploration. Following this, we systematically identified and segregated numerical and categorical variables. The numerical variables comprised various patient demographics and clinical measurements, while the categorical variables included identifiers and binary indicators. Subsequently, we applied standardization to the numerical features using the StandardScaler. This step normalized the numerical variables, transforming them to have a mean of zero and a standard deviation of one. Standardization was crucial for ensuring that all numerical features were on a comparable scale, thereby facilitating the machine learning algorithm's efficiency and performance.

Following standardization, we defined the feature matrix, which included all predictor variables and the target variable, representing the “cognitive decline outcome.”

### Feature Selection

In the feature selection phase, we meticulously included a comprehensive set of variables encompassing demographic, clinical, and laboratory data to predict cognitive decline. The chosen features comprised patient age, sex, smoking status, AH, diabetes status, type of anticoagulation therapy (NOAC or VKA), and various laboratory measurements such as haemoglobin levels, kidney function indicators (eGFR), liver enzymes (AST, ALT, and GGT), and metabolic markers (NT-proBNP).

To address baseline imbalances between patients treated with NOACs and those receiving VKAs, a multivariable logistic regression analysis was conducted. The model incorporated 46 baseline covariates, including demographic variables (e.g., age and sex), clinical characteristics (e.g., hypertension, diabetes, prior stroke, and heart failure), biochemical parameters (e.g., renal function, NT-proBNP, and liver enzymes), composite cardiovascular risk scores (CHA2DS2-VASc and HAS-BLED), and concomitant pharmacological treatments. The aim was to determine whether the type of OAC independently predicted the risk of cognitive decline after adjustment for potential confounding factors.

Even variables that did not exhibit a direct positive correlation with cognitive decline in the preliminary analysis were included. This decision was informed by extensive medical literature, which highlights the complex and multifaceted nature of cognitive decline. By incorporating a broad spectrum of features, including those with less explicit correlations, we aimed to capture the multifaceted interactions and underlying mechanisms contributing to cognitive decline. This approach not only aligns with the established medical literature but also enhances the robustness and comprehensiveness of the predictive model, ensuring that we account for all relevant factors that might influence cognitive outcomes in patients undergoing anticoagulation therapy.

### Model Development and Evaluation

The model development phase involved training a random forest Classifier to predict cognitive decline in patients based on various clinical and demographic features. We initiated this process by splitting the dataset into training and test sets using an 80–20 split, ensuring that the stratification preserved the proportion of cognitive decline instances in both sets. This approach facilitated the evaluation of the model's performance on unseen data. To ensure robustness and generalizability, we employed repeated stratified k-fold cross-validation. Specifically, we used the repeated stratified k-fold method with five splits and ten repeats. This technique provided multiple performance estimates, reducing the variance and offering a more reliable assessment of the model's predictive power. During each cross-validation fold, the model was trained using the training set and evaluated using the validation set. Given the class imbalance observed in the dataset—where approximately 24% of patients experienced cognitive decline—additional strategies were implemented to enhance the model's performance in identifying the minority class. Two configurations of the random forest classifier were evaluated: one employing class weighting and the other trained using the synthetic minority over-sampling technique (SMOTE) applied to the training data.

To further improve sensitivity, the decision threshold for classification was optimized. Specifically, a lower threshold (0.35) was tested in comparison to the default value of 0.5, with the aim of increasing the model's ability to correctly identify true positive cases within the minority class. Key performance metrics, including precision, recall, F1-score, and ROC AUC, were computed to assess the model's performance comprehensively. A confusion matrix was also generated to visualize the model's classification accuracy across different classes.

To assess the individual contribution of predictors, Permutation Importance was applied to the random forest model trained on SMOTE-balanced data and evaluated on the original test set.

## Results

The baseline demographic and clinical characteristics of patients included in the study ( *n* = 983) are reported in Tables 1 to 3 . Their mean age was 76 ± 6 years, 49% were males, 8% were habitual smokers, 86% were affected by AH, and 35% were diabetics. Six hundred and ninety-two patients (70 %) were treated with NOACs (dabigatran: 27 %, rivaroxaban: 38%, apixaban: 24%, and edoxaban: 11%), and the remaining 291 patients (30%) with VKAs.

**Table 1:** Baseline characteristics of patients who completed the study among treatment groups

	Whole population ( *n* = 983)	VKAs ( *n* = 291)	NOACs ( *n* = 692)	*p* -Value
	Demographic and clinical parameters			
Age, y	76 ± 6	77 ± 6	74 ± 5	<0.001
Sex (males), %	49	37	54	<0.001
BMI, kg/m ^2^	29 ± 4	30 ± 3	29 ± 5	0.002
Smokers, %	8	9	7	0.27
Systolic BP, mmHg	133 ± 13	132 ± 10	133 ± 13	0.76
Diastolic BP, mmHg	77 ± 10	77 ± 9	77 ± 10	0.89
Pulse pressure, mmHg	56 ± 12	56 ± 11	56 ± 13	0.68
Atrial function, %				
Paroxysmal	23	25	22	0.36
Persistent	13	14	12	
Permanent	64	61	66	
PM or ICD, %	9	10	8	0.25
CHA2DS2-VASc (score)	4 (3–5)	4 (3–5)	4 (3–5)	0.48
MMSE (score), %				
Normal (>25 MMSE score)	58	62	57	0.11
Borderline (25 MMSE score)	18	14	19	
Moderate cognitive impairment (<25)	24	24	24	
HAS-BLED, %				
Low risk of bleeding	67	74	64	0.004
High risk of bleeding	33	26	36	
Comorbidities				
Arterial hypertension, %	86	82	88	0.02
T2DM, %	35	41	33	0.01
Dyslipidemia, %	37	38	37	0.67
Respiratory failure/COPD, %	35	29	38	0.01
Heart failure, %	33	30	35	0.14
CKD, %	59	58	60	0.57
Sleep apnoea syndrome, %	26	24	27	0.25
Type of SAS, % ( *n* = 257)				
Obstructive sleep apnoea OSA	42	48	40	0.35
Central sleep apnoea CSA	49	46	49	
Mixed sleep apnoea MSA	9	6	11	
Severity of SAS, % ( *n* = 257)				
Mild	96	94	96	0.09
Moderate	3	6	2	
Severe	1	0	2	
Liver disease, %	18	22	17	0.06
Previous stroke or TIA, %	13	9	14	0.02
Previous ACS, %	25	22	26	0.18
ASCVD, %	43	63	35	<0.001

**Table 3:** Baseline characteristics of patients who completed the study among treatment groups

	Biochemical parameters			
	Whole population ( *n* = 983)	VKAs ( *n* = 291)	NOACs ( *n* = 692)	*p* -Value
Albumin, g/dL	3.91 ± 0.36	3.90 ± 0.38	3.92 ± 0.34	0.42
Total cholesterol, mg/dL	167 ± 42	165 ± 41	168 ± 43	0.33
LDL cholesterol, mg/dL	100 ± 35	98 ± 34	101 ± 35	0.21
HDL cholesterol, mg/dL	51 ± 16	52 ± 16	51 ± 16	0.12
Triglycerides, mg/dL	100 (74–136)	95 (70–132)	101 (76–138)	0.05
Haemoglobin, g/dL	13.36 ± 1.51	13.68 ± 0.99	13.23 ± 1.67	<0.001
PLT, × 10 ^3^ /uL	219 ± 60	219 ± 60	218 ± 60	0.82
Na, mmol/L	141 (139–142)	141 (139–142)	141 (139–142)	0.21
K, mmol/L	4.4 (4.2–4.7)	4.5 (4.2–4.7)	4.4 (4.1–4.7)	0.012
Creatinine, mg/dL	1.05 ± 0.31	1.06 ± 0.35	1.05 ± 0.29	0.69
e-GFR, mL/min/1.73 m ^2^	64 ± 18	64 ± 20	64 ± 17	0.98
Clearance creatinine, mL/min	68 ± 23	71 ± 24	67 ± 22	0.002
NT-proBNP, pg/mL	562 (512–1,357)	598 (523–1,737)	562 (489–1,356)	0.025
Fasting glucose, mg/dL	103 (95–119)	106 (99–113)	102 (94–126)	0.21
Fasting insulin, μU/mL	16.14 ± 6.62	15.85 ± 6.24	16.26 ± 6.78	0.37
HOMA	4.22 (2.87–5.75)	3.90 (2.79–5.34)	4.32 (2.93–5.88)	0.046
Uric acid, mg/dL	5.5 (4.9–6.3)	5.4 (4.9–6.3)	5.5 (5.1–6.3)	0.41
AST, IU/L	20 (16–24)	19 (16–23)	20 (17–25)	0.0002
ALT, IU/L	19 (13–25)	17 (13–24)	19 (14–26)	0.013
Alkaline phosphatase, IU/L	78 (66–107)	76 (66–106)	78 (66–110)	0.20
GGT, IU/L	32 (21–42)	32 (19–42)	32 (21–42)	0.77

At baseline, 58% of patients had a normal value of MMSE score, 18% had a borderline, and the remaining 24% had mild CoI. At baseline, the MMSE did not differ between the two groups (NOAC vs. VKA, *p* = 0.11). Patients treated with NOACs were younger (74 ± 5 vs. 77 ± 6 years, *p* < 0.001), had a higher percentage of males (54% vs. 37%, *p* < 0.001), and had a lower BMI (29 ± 5 vs. 30 ± 3 kg/m ^2^ , *p* = 0.002) compared with those on VKAs.

Patients on NOACs displayed a higher prevalence of AH (88% vs. 82%, *p* = 0.02), respiratory failure/COPD (38% vs. 29%, *p* = 0.01) and a lower percentage of T2DM (33% vs. 41%, *p* = 0.01) as compared with those on VKAs as well as a lower prevalence of patients with background cardiovascular comorbidities (previous stroke or TIA 14% vs. 9%, *p* = 0.02, atherosclerotic cardiovascular disease (ASCVD) 35% vs. 63%, *p* < 0.001; CHA2DS2-VASc score: 4 (3–5) versus 4 (3–5), *p* = 0.48; HAS-BLED: low risk of bleeding 64% versus 74% and high risk of bleeding 36% versus 26%, *p* = 0.004.

Compared with patients on VKAs, those on NOACs were also less frequently treated with PPI (76% vs. 86%, *p* = 0.001), ACEi/ARBs (75% vs. 81%, *p* = 0.03), β-blockers (57% vs. 67%, *p* = 0.005), anti-arrhythmic drugs (15% vs. 20%, *p* = 0.04), GLP1-RAs (29% vs. 40%, *p* = 0.001), lipid-lowering drugs (43% vs. 52%, *p* = 0.02) and previous antiplatelet therapy (30% vs. 65%, *p* < 0.001). There were no differences for SGLT2i treatment (20% vs. 25%, *p* = 0.12; Table 2 ).

**Table 2:** Baseline characteristics of patients who completed the study among treatment groups

	Whole population ( *n* = 983)	VKAs ( *n* = 291)	NOACs ( *n* = 692)	*p* -Value
PPIs, %	79	86	76	0.001
Nitrates, %	6	4	7	0.09
ACEi/ARBs, %	77	81	75	0.03
β-blockers, %	60	67	57	0.005
Digitalis, %	15	13	16	0.18
Calcium channel blockers, %	15	15	15	0.94
MRAs, %	23	23	24	0.73
ARNI, %	26	25	26	0.74
Anti-arrhythmics drugs, %	17	20	15	0.04
Type of AAD drugs, %				
None	83	80	85	0.05
Amiodarone	6	5	6	
Flecainide	6	8	4	
Propafenone	4	5	4	
Other AAD drugs	1	2	1	
Metformina/OADs, %	23	21	23	0.47
Insulin, %	11	10	12	0.43
SGLT2i, %	22	25	20	0.12
GLP1-RAs, %	32	40	29	0.001
Lipid-lowering drugs, %	46	52	43	0.02
Previous antiplatelet therapy, %	40	65	30	<0.001

Patients on NOACs had lower levels of Haemoglobin, Kaliemia, eGFR, NT-proBNP, and higher values of Homeostatic Model Assessment (HOMA), AST, ALT, and GGT ( Table 3 ).

In the multivariable logistic regression model, treatment with NOACs was significantly associated with a reduced likelihood of cognitive decline compared with VKAs (odds ratio: 0.322; 95% confidence interval [CI]: 0.221–0.469; *p* < 0.0001), corresponding to 67.8% lower adjusted odds of cognitive decline in the NOAC group. To validate these findings, the same set of covariates was incorporated into the random forest classifier.

During the follow-up period, the exploratory analysis of all-cause mortality across the treatment groups revealed that the mortality rate among patients treated with NOACs was 24.9% (178/714), while the rate among those treated with VKAs was 25.0% (86/291). A chi-square test showed no statistically significant difference between the groups ( *χ* ^2^ = 2.049, *p* = 0.152).

The random forest classifier demonstrated strong and consistent performance in predicting cognitive decline. During repeated stratified cross-validation, the model achieved a mean ROC AUC of 0.8715, with a standard deviation of 0.0273, indicating high stability across different data partitions ( Fig. 2 ). On the held-out test set, the class-weighted configuration yielded a ROC AUC of 0.880, a precision of 0.94, and a recall of 0.47 for the cognitive decline class.

**Fig. 2:**
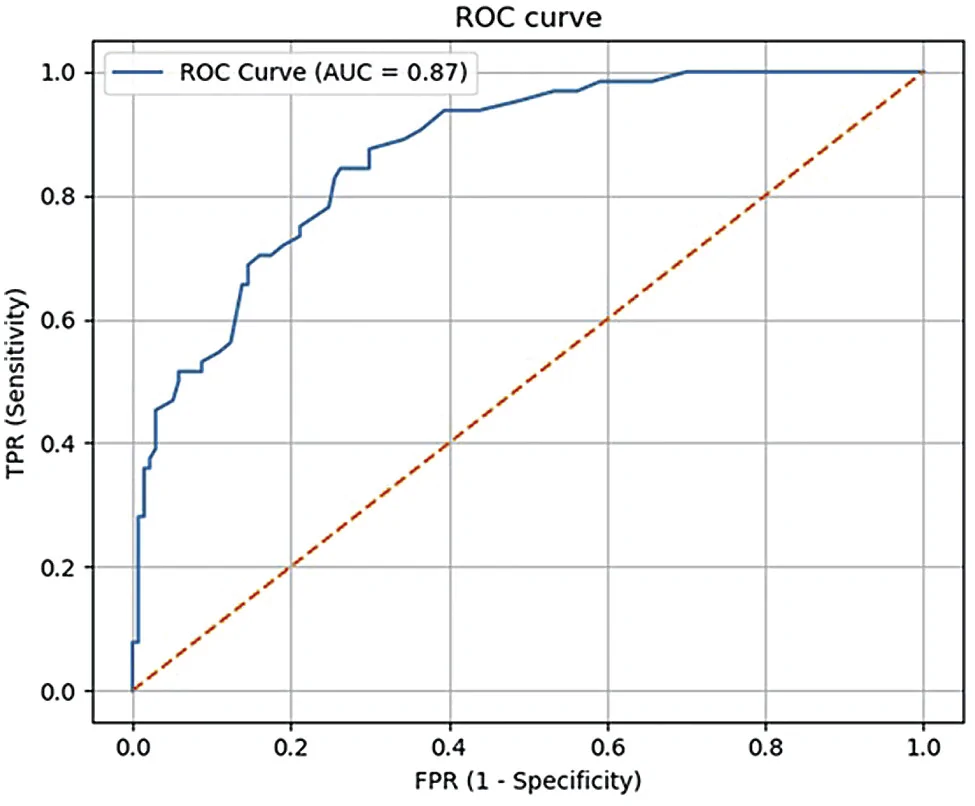
Receiver Operating Characteristic (ROC) curve for the random forest model. The curve illustrates the trade-off between sensitivity (true positive rate) and 1-specificity (false positive rate) across different classification thresholds. The area under the curve (AUC) is 0.87, indicating high discriminative ability of the model in distinguishing patients with and without cognitive decline. The diagonal line represents a no-skill classifier (AUC = 0.5) for reference.

An alternative configuration trained using the SMOTE achieved a slightly lower ROC AUC of 0.869 but improved recall to 0.53, with a comparable F1-score of 0.62 and a reduction in precision to 0.74. To further enhance sensitivity for the minority class, the classification threshold was lowered to 0.35. This adjustment increased recall to 0.84 and F1-score to 0.68, while precision declined to 0.57 and overall accuracy to 75%.

The model's discriminative ability under class imbalance was further supported by the precision–recall AUC, which reached 0.763 ( Fig. 3 ).

**Fig. 3:**
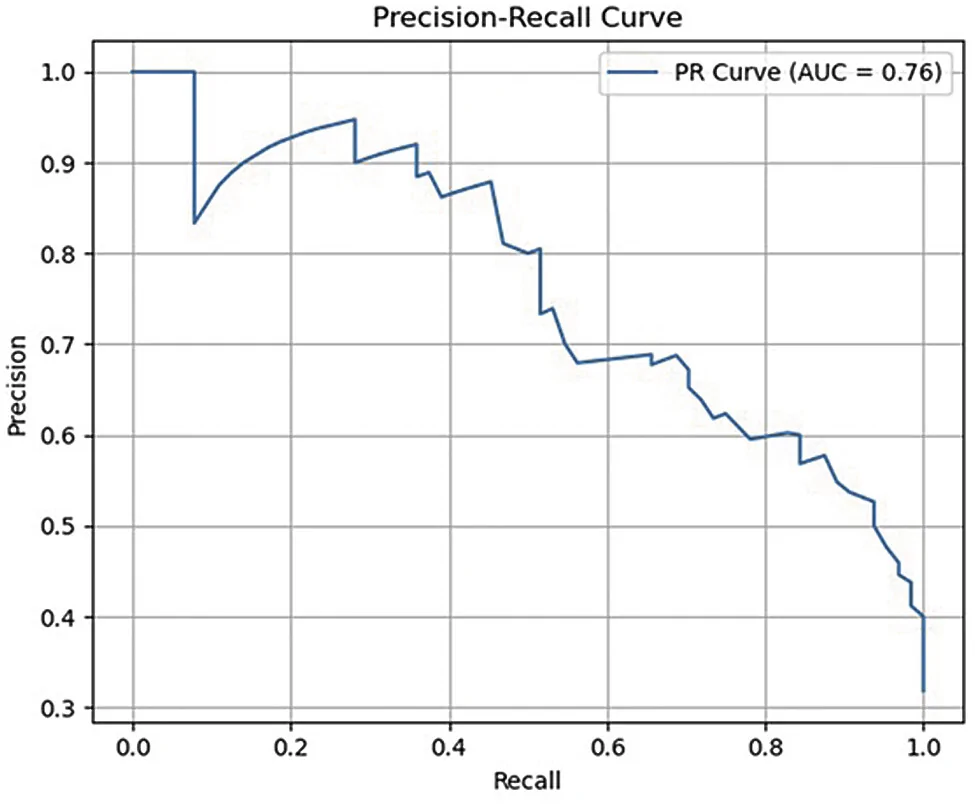
Precision–recall (PR) curve for the random forest model. This curve shows the relationship between precision and recall across various classification thresholds. The Area Under the Curve (PR AUC) is 0.76, reflecting the model's ability to identify true positive cases of cognitive decline in the context of class imbalance. A higher PR AUC indicates better performance in correctly identifying patients at risk, particularly when the positive class (cognitive decline) is underrepresented.

The confusion matrix showed 125 true negatives, 30 false negatives, 12 false positives, and 34 true positives ( Fig. 4 ).

**Fig. 4:**
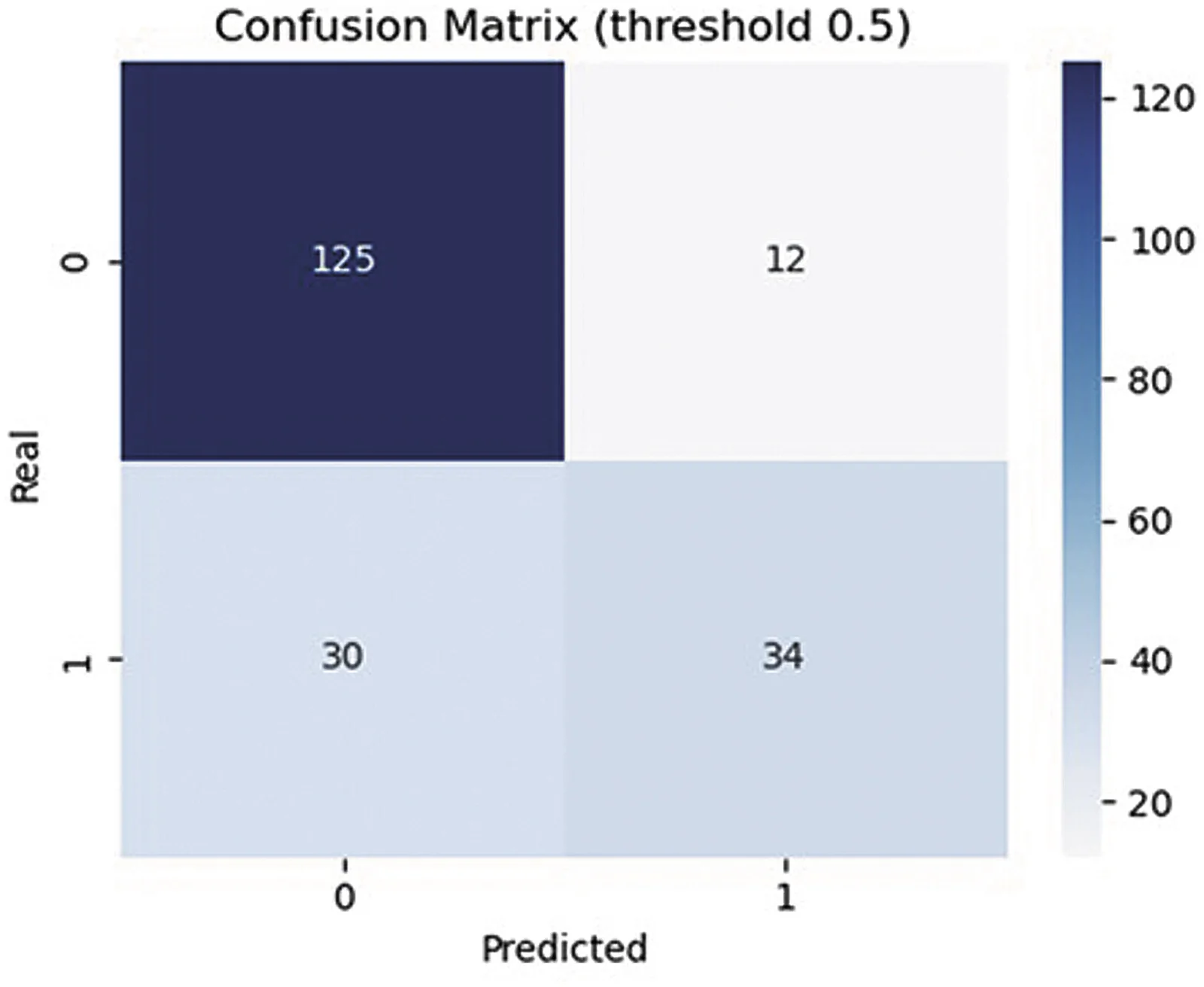
Confusion matrix of the random forest classifier using a threshold of 0.5. The matrix summarizes model predictions on the test set. The classifier correctly identified 125 patients without cognitive decline (true negatives) and 34 with cognitive decline (true positives) but misclassified 30 patients with cognitive decline as nondecline (false negatives), and 12 patients without decline as decline (false positives). These results correspond to a recall of 0.53 and a precision of 0.74 for the decline class.

Moreover, the analysis of predicted probabilities revealed a significant difference between treatment groups. The mean predicted probability of cognitive decline was 0.50 (standard deviation [SD]: 0.37) in patients treated with VKAs and 0.24 (SD: 0.30) in those treated with NOACs. A Kolmogorov–Smirnov test confirmed a statistically significant difference in the distribution of predicted probabilities (KS statistic = 0.385, *p* < 0.001), indicating a consistent association between anticoagulant type and cognitive risk across all model configurations ( Fig. 5 ).

**Fig. 5:**
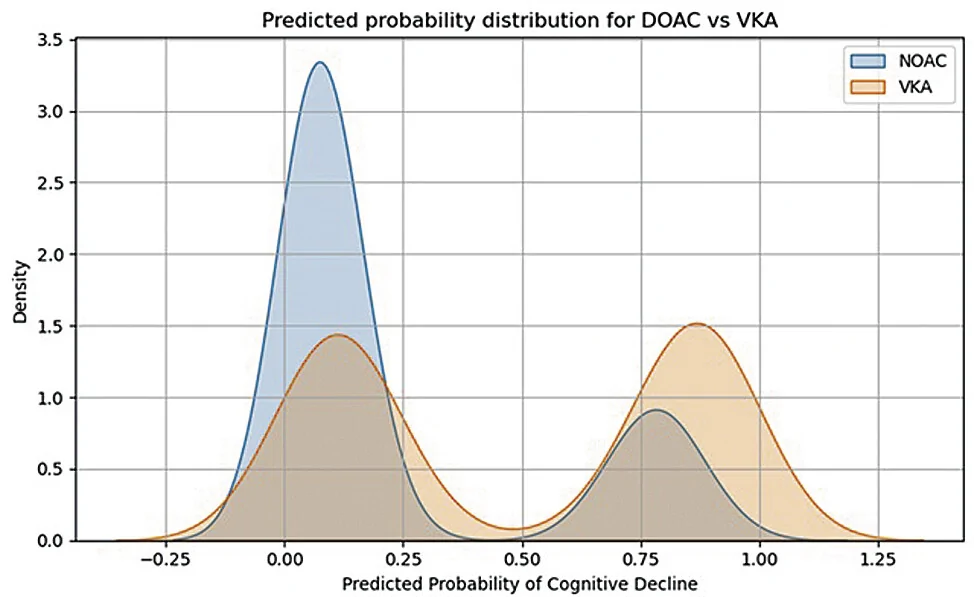
Kernel density estimation of predicted probabilities of cognitive decline for NOAC- and VKA-treated patients. The plot displays the distribution of predicted probabilities from the RANDOM FOREST model for the two treatment groups. Patients on VKAs exhibit a right-skewed distribution with higher predicted probabilities of cognitive decline, while those on NOACs show a left-skewed distribution with lower predicted risk. This separation is statistically confirmed by a Kolmogorov–Smirnov test (KS statistic = 0.385, *p* < 0.001), indicating a significant difference in model predictions between groups.

The Permutation Importance analysis identified “OAT” as the most influential predictor of cognitive decline (mean importance = 0.042), substantially exceeding all other variables. The next most relevant features included glycemia (0.018), systolic blood pressure (0.013), BMI (0.009), albumin (0.009), and potassium (0.008). A table summarizing the top 15 predictors is reported in Fig. 6 .

**Fig. 6:**
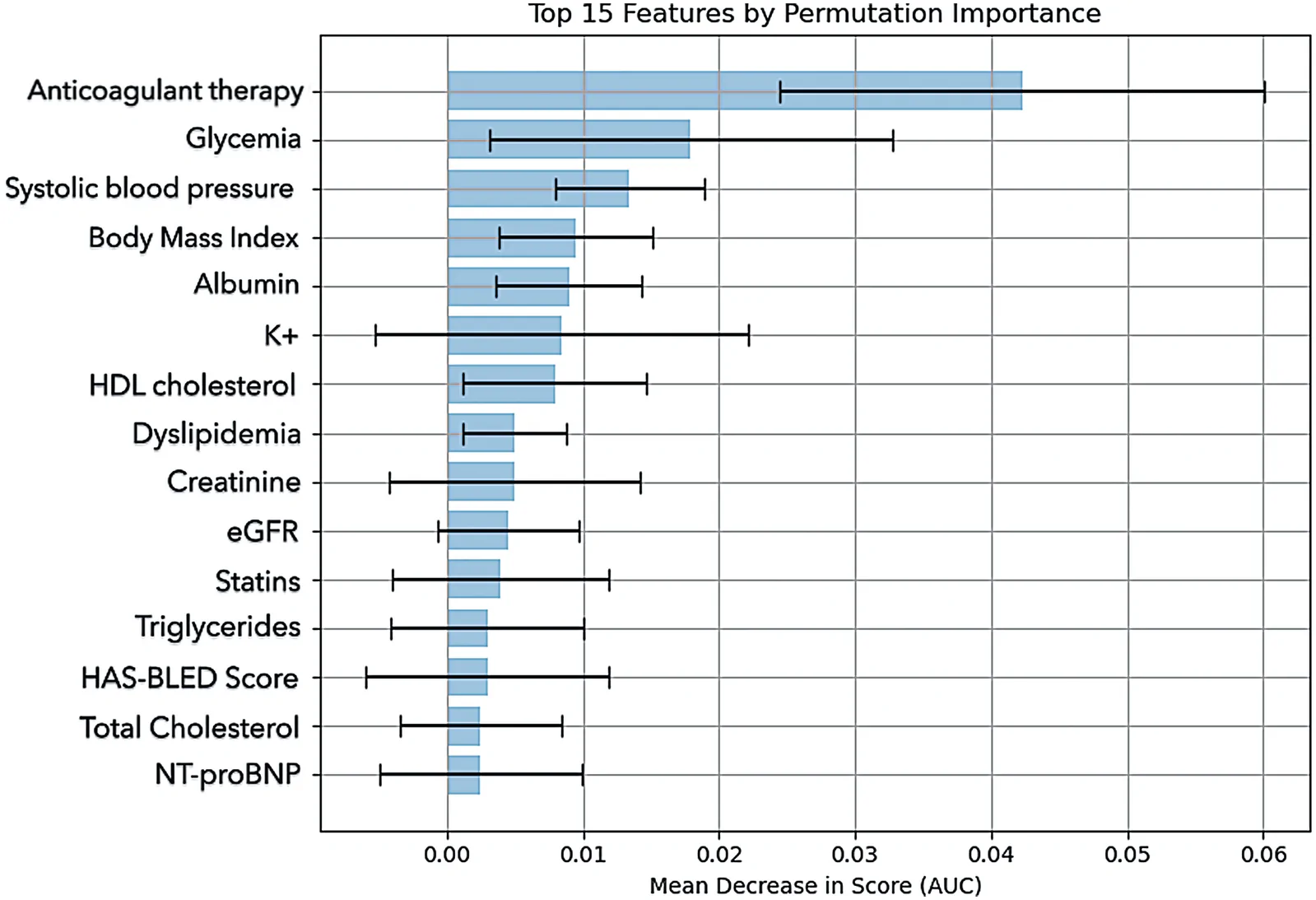
Top 15 predictors of cognitive decline ranked by permutation importance. This bar plot displays the mean decrease in ROC AUC associated with randomly permuting each feature in the SMOTE-balanced random forest model, evaluated on the original test set. Error bars represent the standard deviation across 10 permutations. The variable “anticoagulant therapy” emerged as the most influential predictor, followed by glycemia, systolic blood pressure, BMI, albumin, and potassium levels. This analysis confirms the dominant role of anticoagulant type in predicting cognitive outcomes, even within a multivariable and nonlinear framework.

## Discussion

The results of this study highlight the efficacy of the random forest classifier in predicting cognitive decline among patients undergoing anticoagulation therapy. The model demonstrates robust and consistent performance with a mean ROC AUC of 0.8719 across cross-validation folds and a standard deviation of 0.0270. The high ROC AUC score of 0.8804 on the test set further corroborates the model's capability to distinguish between patients who will and will not experience cognitive decline. These metrics underscore the model's reliability, making it a valuable tool for clinical decision-making.

The random forest classifier was chosen for this study due to several key advantages it offers in the context of clinical data analysis. First, random forest is particularly well-suited to handle complex and diverse datasets, such as those used in this study, which contain a mixture of clinical, demographic, and laboratory variables. The algorithm efficiently processes both numerical and categorical variables, making it a versatile tool for clinical datasets where features may have varying relationships with the target variable, in this case, cognitive decline.

One of the significant strengths of the random forest algorithm is its robustness to noisy or less important features. In this study, several variables were included based on their potential relevance to cognitive outcomes, even when they did not show strong correlations with cognitive decline in the preliminary analysis. The random forest naturally reduces the impact of these less informative features through its inherent ability to assign lower importance to variables that do not significantly contribute to the prediction without compromising model performance.

Another critical advantage is the algorithm's ability to reduce overfitting, particularly when working with medical datasets that are prone to variability and noise. The use of “bagging (bootstrap aggregation),” where multiple decision trees are trained on random subsets of the data, mitigates the risk of overfitting by aggregating the predictions of individual trees. This process ensures that the model generalizes well to new, unseen data, as demonstrated by the robust performance metrics observed during cross-validation and testing.

Moreover, random forest is inherently robust in handling imbalanced datasets, a common challenge in medical studies where one outcome (e.g., cognitive decline) is less frequent than its counterpart (e.g., no cognitive decline). Although this study involved balancing techniques like stratification, the algorithm's ability to handle such imbalances ensured that both cognitive and noncognitive decline cases were effectively identified.

Finally, the scalability and flexibility of random forests make it ideal for datasets with large feature spaces, such as the one used in this study. The ability to handle large datasets with numerous features while maintaining computational efficiency underscores the suitability of random forest for clinical applications where both the volume and complexity of data can be substantial.

The actual effects of direct oral anticoagulant (DOAC) versus VKA therapy on cognitive decline burden in patients with AF are not yet fully clear.

In our study, the analysis of predicted probabilities of cognitive decline revealed that the mean predicted probability for patients on VKA was 0.5027 with a standard deviation of 0.3807, while for those on NOAC, it was 0.2473 with a standard deviation of 0.3043. The Kolmogorov–Smirnov test results indicated a KS statistic of 0.3604 and a *p* -value < 0.001, suggesting a significant difference in the distribution of predicted probabilities between the two groups.

A recent study 20 estimated dementia incidence in patients with newly diagnosed AF and taking an anticoagulant, specifically the use of DOACs. A total of 1,083,338 beneficiaries were included in the study, 58.5% female, with a mean (SD) age of 77.2 (6.75) years. Among anticoagulated, incident AF cohorts, the use of DOACs increased from 10.6% in their first year of availability (2011) to 41.4% in 2017. Among incident AF cohorts taking any OAC, 3-year dementia incidence did not change significantly over the cohorts after adjusting for confounders. The authors concluded that the Increased use of DOACs among incident AF cohorts from 2007 to 2017 was not associated with significant declines in dementia or stroke risk. Consideration of similar stroke and dementia risk, as well as differences in cost, is warranted when weighing the risks and benefits of available OACs.

Another study 21 assessed the risk of new all-cause dementia and vascular dementia in AF patients treated with either DOAC or VKAs. In this study, the authors evaluated anonymized electronic medical records. AF patients treated with DOACs within 1 month of AF diagnosis were 1:1 propensity score-matched with those treated with a VKA. Among patients aged 65 to 74 years who were followed, DOAC treatment was associated with a lower risk of dementia compared with VKAs (hazards ratio [HR]: 0.72; 95% CI: 0.59–0.86). Among patients who completed 10 years of follow-up after propensity score matching, the final cohort consisted of 19,208 well-matched AF patients. All-cause dementia was diagnosed in 314 (3.3%) patients among those treated with DOACs and 451 (4.7%) among the VKA-treated patients. DOAC treatment was associated with a significantly lower risk of all-cause dementia during a 10-year follow-up period compared with VKA treatment (HR: 0.72; 95% CI: 0.62–0.83). This propensity-score matched analysis showed that among AF patients, treatment with DOACs for a period of 10 years was associated with a lower risk of all-cause dementia and vascular dementia compared with VKA treatment, an effect that was not apparent in those treated for a shorter duration.

A crucial factor to consider in patients with AF is patient frailty. AF patients who are frail have been reported to exhibit greater cognitive decline and lower baseline cognitive function compared with nonfrail patients. 22 While our findings suggest that NOAC therapy is associated with better cognitive outcomes than VKA therapy, this observation could be influenced by confounding factors. The groups in our study differed in age and comorbidities (with VKA patients being older and having more comorbid conditions), which are elements of frailty. Indeed, frailty itself may predispose patients to CoI, regardless of the anticoagulant type. Moreover, a recent randomized trial (FRAIL-AF) showed that switching frail elderly AF patients from warfarin to an NOAC did not improve clinical outcomes and was associated with increased bleeding complications. 23

This underscores that any cognitive benefit of NOACs must be weighed against individual patient factors like frailty and that anticoagulation strategies should be personalized. This underscores the importance of a holistic “patient-centered” approach to the patient with AF.

Systematically reviewing large EHR data collected across patients' entire visit history within a health care system can facilitate early detection of cognitive decline by identifying when patients first reported signs or symptoms of cognitive decline to any health care professional.

However, despite the overall strong performance, this study has notable weaknesses that warrant discussion. Current approaches to identifying cognitive decline from the EHR have limitations. These approaches commonly rely on billing codes or medications and are likely to be insensitive. Prior studies primarily focused on the stages of cognitive decline from MCI to dementia.

Research on detecting early cognitive decline preceding MCI and based on unstructured EHRs is limited. 24

The analysis of predicted probabilities also revealed a significant difference between patients on VKA and those on NOAC, as indicated by the Kolmogorov–Smirnov test (KS statistic: 0.385, *p* -value < 0.001; Fig. 4 ). The higher mean predicted probability of cognitive decline for patients on VKA (0.50) compared with those on NOAC (0.24) suggests a potential association between the type of anticoagulation therapy and cognitive outcomes.

These findings are reinforced by the Permutation Importance analysis, which demonstrated that the type of OAT was the most impactful feature in the random forest model. The robustness of this association, in a multivariable and nonlinear context, suggests that the observed effect is unlikely to be confounded by other clinical characteristics.

Moreover, the convergence of findings from both traditional regression analysis and machine learning approaches reinforces the robustness of the association between NOAC therapy and reduced cognitive decline. The logistic regression confirmed the independent predictive value of anticoagulant type after controlling for a broad range of baseline variables, while the random forest classifier—despite its nonparametric nature—identified the same variable as a top predictor. This methodological triangulation strengthens the credibility of the observed association and reduces the likelihood that it is driven by confounding or model-specific artifacts.

Nevertheless, the possible pleiotropic effects of DOACs, such as antioxidant, anti-inflammatory, anti-fibrotic, and anti-senescence effects, as well as decreased permeability, may provide a partial answer to these questions.

A recent study demonstrated that in a population of patients with HF and AF, patients treated with VKAs or DOACs are distinguishable according to features related to inflammation, endothelial function, cardiac remodeling, and clinical status. 25

It is conceivable that the actions of DOACs can potentially extend beyond their conventional role in anticoagulation as inhibitors of the blood coagulation factors thrombin and fXa. Indeed, it is well established that these proteases mediate several (patho)physiological processes such as inflammation, atherothrombosis, and angiogenesis 26 by triggering the activation of proteinase-activated receptors (PARs). The ability of DOACs to regulate PAR responses provides new insights into their actions beyond anticoagulation. Indeed, several preclinical studies have demonstrated that DOACs exhibit pleiotropic actions on endothelial cells, including anti-inflammatory, anti-atherosclerotic, and anti-fibrotic effects, as well as the preservation of endothelial integrity. 27 28 29 It has been established that DOACs block specific pro-inflammatory processes, regulating the expression of key cytokines in various in vitro cell systems as well as in animal models of atherosclerotic lesions. 30 Furthermore, the decrease in endothelial permeability and reduction in ROS generation have been reported as primary mechanisms underlying the capability of DOACs to enhance endothelial barrier integrity. 31

Lastly, it is important to acknowledge that the MMSE, although widely used in clinical settings, has limited sensitivity for detecting dementia in its early stages. Individuals in the prodromal phase of cognitive decline may score within the normal range despite clinically meaningful impairments, potentially leading to an underestimation of cognitive dysfunction. 32 Nevertheless, the MMSE offers several notable advantages. It is a brief, easy-to-administer, and well-validated tool that provides a general overview of key cognitive domains. Its widespread use and clinician familiarity enhance its utility in routine practice. To address its limitations, future research may benefit from incorporating more sensitive screening instruments, such as the Montreal Cognitive Assessment, which can better detect subtle cognitive deficits in early dementia.

## Conclusion

The model's high precision and overall accuracy are offset by its lower recall for the cognitive decline class, highlighting the need for continued refinement. Despite these limitations, the significant differences in predicted probabilities between therapy types provide valuable insights that could guide future research and clinical practice. Future prospective studies and validation in an external court will be needed to validate the predictive performance of our model on CoI in patients with AF in relation to treatment. Future studies should focus on enhancing the model's recall and addressing potential biases to fully realize the potential of machine learning in predicting cognitive decline in patients undergoing anticoagulation therapy.

Our study highlighted that DOAC treatment, compared with VKAs, adjusted for potential confounding factors, is the main factor influencing a reduction in CoI in a population of elderly patients with AF. The incidence of CoI and dementia represents one of the most important factors influencing patients' quality of life. The results of this study, therefore, suggest implementing awareness of this outcome in the complex choice of the best anticoagulant treatment in the elderly population.

## Notes

**What is known about this topic?**

- Thromboembolic events clearly contribute to CoI in AF patients, highlighting the importance of preventing such events through OAT.
- AF is associated with cognitive decline and dementia even in the absence of a clinically significant stroke.
- NOACs have a better safety profile and equivalent efficacy compared with VKAs. However, the literature shows no net cognitive function benefit of NOACs over VKAs.

**What does this paper add?**

- Machine learning lies in its capacity to discern patterns and correlations within complex datasets, yielding valuable insights into disease diagnosis, prognosis, and treatment.
- Our machine learning model has, therefore, demonstrated a good predictive power in predicting cognitive decline, indicated by an AUC value of 0.88, a good accuracy documented by a precision greater than 80% in the precision–recall curve, a good sensitivity and specificity assessed through the Kolmogorov–Smirnov test.
- Our study demonstrates a significant advantage of the use of DOACs compared with VKAs in preventing cognitive decline in patients with AF, especially in the elderly population.

## References

[1] Miyasaka Y Barnes M E Gersh B J Secular trends in incidence of atrial fibrillation in Olmsted County, Minnesota, 1980 to 2000, and implications on the projections for future prevalence Circulation 2006 114 02 119 125 10.1161/CIRCULATIONAHA.105.595140 16818816

[2] Van Gelder I C Rienstra M Bunting K V ESC Scientific Document Group 2024 ESC Guidelines for the management of atrial fibrillation developed in collaboration with the European Association for Cardio-Thoracic Surgery (EACTS) Eur Heart J 2024 45 36 3314 3414 10.1093/eurheartj/ehae176 39210723

[3] Joglar J A Chung M K Armbruster A L Peer Review Committee Members 2023 ACC/AHA/ACCP/HRS guideline for the diagnosis and management of atrial fibrillation: a report of the American College of Cardiology/American Heart Association Joint Committee on clinical practice guidelines Circulation 2024 149 01 e1 e156 10.1161/CIR.0000000000001193 PMC11095842 38033089

[4] Wang Y Guo Y Qin M Expert Reviewers 2024 Chinese Expert Consensus guidelines on the diagnosis and treatment of atrial fibrillation in the elderly, endorsed by Geriatric Society of Chinese Medical Association (Cardiovascular Group) and Chinese Society of Geriatric Health Medicine (Cardiovascular Branch): executive summary Thromb Haemost 2024 124 10 897 911 10.1055/a-2325-5923 PMC11436293 38744425

[5] Kalantarian S Ruskin J N Atrial fibrillation and cognitive decline: phenomenon or epiphenomenon? Cardiol Clin 2016 34 02 279 285 10.1016/j.ccl.2015.12.011 27150176

[6] Thacker E L McKnight B Psaty B M Atrial fibrillation and cognitive decline: a longitudinal cohort study Neurology 2013 81 02 119 125 10.1212/WNL.0b013e31829a33d1 PMC3770176 23739229

[7] Bunch T J Weiss J P Crandall B G Atrial fibrillation is independently associated with senile, vascular, and Alzheimer's dementia Heart Rhythm 2010 7 04 433 437 10.1016/j.hrthm.2009.12.004 20122875

[8] Vermeer S E Prins N D den Heijer T Hofman A Koudstaal P J Breteler M M Silent brain infarcts and the risk of dementia and cognitive decline N Engl J Med 2003 348 13 1215 1222 10.1056/NEJMoa022066 12660385

[9] Poggesi A Inzitari D Pantoni L Atrial fibrillation and cognition: epidemiological data and possible mechanisms Stroke 2015 46 11 3316 3321 10.1161/STROKEAHA.115.008225 26396028

[10] Diener H-C Hart R G Koudstaal P J Lane D A Lip G YH Atrial fibrillation and cognitive function: JACC review topic of the week J Am Coll Cardiol 2019 73 05 612 619 10.1016/j.jacc.2018.10.077 30732716

[11] Jefferson A L Beiser A S Himali J J Low cardiac index is associated with incident dementia and Alzheimer disease: the Framingham Heart Study Circulation 2015 131 15 1333 1339 10.1161/CIRCULATIONAHA.114.012438 PMC4398627 25700178

[12] Stefansdottir H Arnar D O Aspelund T Atrial fibrillation is associated with reduced brain volume and cognitive function independent of cerebral infarcts Stroke 2013 44 04 1020 1025 10.1161/STROKEAHA.12.679381 PMC3632359 23444303

[13] Knecht S Oelschläger C Duning T Atrial fibrillation in stroke-free patients is associated with memory impairment and hippocampal atrophy Eur Heart J 2008 29 17 2125 2132 10.1093/eurheartj/ehn341 18667399

[14] Roberti R Iannone L F Palleria C Direct oral anticoagulants: from randomized clinical trials to real-world clinical practice Front Pharmacol 2021 12 684638 10.3389/fphar.2021.684638 PMC8188985 34122113

[15] Armentaro G D'Arrigo G Bo M Medium-term and long-term renal function changes with direct oral anticoagulants in elderly patients with atrial fibrillation Front Pharmacol 2023 14 1.21056E6 10.3389/fphar.2023.1210560 PMC10352777 37469870

[16] Mongkhon P Fanning L Lau W CY Oral anticoagulant and reduced risk of dementia in patients with atrial fibrillation: a population-based cohort study Heart Rhythm 2020 17(Pt A):706 713 10.1016/j.hrthm.2020.01.007 31931172

[17] Armentaro G D'Arrigo G Pastori D Long-term cognitive function changes with non-vitamin K oral anticoagulants in older patients with atrial fibrillation. a multicenter cohort study Eur J Clin Invest 2024 55 01 e14321 10.1111/eci.14321 39311651

[18] Daidone M Ferrantelli S Tuttolomondo A Machine learning applications in stroke medicine: advancements, challenges, and future prospectives Neural Regen Res 2024 19 04 769 773 10.4103/1673-5374.382228 PMC10664112 37843210

[19] Zhou L Pan S Wang J Vasilakos A V Machine learning on big data: opportunities and challenges Neurocomputing 2017 237 350 361

[20] Thunell J Wood K Wharton W Joyce G Ferido P Zissimopoulos J Population dementia incidence and direct oral anticoagulant use in a representative population with atrial fibrillation Neurology 2024 103 01 e209568 10.1212/WNL.0000000000209568 PMC11226323 38857466

[21] Sagris D Ntaios G Buckley B JR Direct oral anticoagulants are associated with lower risk of dementia in patients with atrial fibrillation Eur J Intern Med 2024 121 114 120 10.1016/j.ejim.2023.10.033 37914656

[22] Polidoro A Stefanelli F Ciacciarelli M Pacelli A Di Sanzo D Alessandri C Frailty in patients affected by atrial fibrillation Arch Gerontol Geriatr 2013 57 03 325 327 10.1016/j.archger.2013.04.014 23706973

[23] Joosten L PT van Doorn S van de Ven P M Safety of switching from a vitamin K antagonist to a non-vitamin K antagonist oral anticoagulant in frail older patients with atrial fibrillation: results of the FRAIL-AF randomized controlled trial Circulation 2024 149 04 279 289 10.1161/CIRCULATIONAHA.123.066485 37634130

[24] Wang L Laurentiev J Yang J Development and validation of a deep learning model for earlier detection of cognitive decline from clinical notes in electronic health records JAMA Netw Open 2021 4 11 e2135174 10.1001/jamanetworkopen.2021.35174 PMC8603078 34792589

[25] Mele M Mele A Imbrici P Pleiotropic effects of direct oral anticoagulants in chronic heart failure and atrial fibrillation: machine learning analysis Molecules 2024 29 11 2651 10.3390/molecules29112651 PMC11174118 38893525

[26] Danckwardt S Hentze M W Kulozik A E Pathologies at the nexus of blood coagulation and inflammation: thrombin in hemostasis, cancer, and beyond J Mol Med (Berl) 2013 91 11 1257 1271 10.1007/s00109-013-1074-5 PMC3825489 23955016

[27] Álvarez E Paradela-Dobarro B Raposeiras-Roubín S González-Juanatey J R Protective, repairing and fibrinolytic effects of rivaroxaban on vascular endothelium Br J Clin Pharmacol 2018 84 02 280 291 10.1111/bcp.13440 PMC5777430 28940408

[28] Torramade-Moix S Palomo M Vera M Apixaban downregulates endothelial inflammatory and prothrombotic phenotype in an in vitro model of endothelial dysfunction in uremia Cardiovasc Drugs Ther 2021 35 03 521 532 10.1007/s10557-020-07010-z 32651897

[29] Imano H Kato R Nomura A Rivaroxaban attenuates right ventricular remodeling in rats with pulmonary arterial hypertension Biol Pharm Bull 2021 44 05 669 677 10.1248/bpb.b20-01011 33612567

[30] Zhou Q Bea F Preusch M Evaluation of plaque stability of advanced atherosclerotic lesions in apo E-deficient mice after treatment with the oral factor Xa inhibitor rivaroxaban Mediators Inflamm 2011 2011 432080 10.1155/2011/432080 PMC3134269 21772662

[31] Zolotoff C Puech C Roche F Perek N Effects of intermittent hypoxia with thrombin in an in vitro model of human brain endothelial cells and their impact on PAR-1/PAR-3 cleavage Sci Rep 2022 12 01 12305 10.1038/s41598-022-15592-x PMC9296553 35853902

[32] Arevalo-Rodriguez I Smailagic N Roqué-Figuls M Mini-mental state examination (MMSE) for the early detection of dementia in people with mild cognitive impairment (MCI) Cochrane Database Syst Rev 2021 7 07 CD010783 10.1002/14651858.CD010783.pub3 PMC8406467 34313331

